# Limited hydraulic recovery in seedlings of six tree species with contrasting leaf habits in subtropical China

**DOI:** 10.3389/fpls.2022.967187

**Published:** 2022-08-11

**Authors:** Honglang Duan, Defu Wang, Nan Zhao, Guomin Huang, Víctor Resco de Dios, David T. Tissue

**Affiliations:** ^1^Institute for Forest Resources and Environment of Guizhou, Key Laboratory of Forest Cultivation in Plateau Mountain of Guizhou Province, College of Forestry, Guizhou University, Guiyang, China; ^2^Jiangxi Provincial Key Laboratory for Restoration of Degraded Ecosystems and Watershed Ecohydrology, Nanchang Institute of Technology, Nanchang, China; ^3^Key Laboratory of Vegetation Restoration and Management of Degraded Ecosystems, Guangdong Provincial Key Laboratory of Applied Botany, South China Botanical Garden, Chinese Academy of Sciences, Guangzhou, China; ^4^School of Life Science and Engineering, Southwest University of Science and Technology, Mianyang, China; ^5^Department of Crop and Forest Sciences, University of Lleida, Lleida, Spain; ^6^Joint Research Unit CTFC-AGROTECNIO-CERCA Center, Lleida, Spain; ^7^Hawkesbury Institute for the Environment, Western Sydney University, Penrith, NSW, Australia; ^8^Global Centre for Land-Based Innovation, Western Sydney University, Richmond, NSW, Australia

**Keywords:** gas exchange, water relations, hydraulic conductivity, recovery, xylem embolism, NSC

## Abstract

Subtropical tree species may experience severe drought stress due to variable rainfall under future climates. However, the capacity to restore hydraulic function post-drought might differ among co-occurring species with contrasting leaf habits (e.g., evergreen and deciduous) and have implications for future forest composition. Moreover, the links between hydraulic recovery and physiological and morphological traits related to water-carbon availability are still not well understood. Here, potted seedlings of six tree species (four evergreen and two deciduous) were grown outdoors under a rainout shelter. They grew under favorable water conditions until they were experimentally subjected to a soil water deficit leading to losses of *ca.* 50% of hydraulic conductivity, and then soils were re-watered to field capacity. Traits related to carbon and water relations were measured. There were differences in drought responses and recovery between species, but not as a function of evergreen or deciduous groups. *Sapindus mukorossi* exhibited the most rapid drought response, which was associated with a suite of physiological and morphological traits (larger plant size, the lowest hydraulic capacitance (*C*_branch_), higher minimum conductance (*g*_min_) and lower *HV* (Huber value)). Upon re-watering, xylem water potential exhibited fast recovery in 1–3 days among species, while photosynthesis at saturating light (*A*_sat_) and stomatal conductance (*g*_s_) recovery lagged behind water potential recovery depending on species, with *g*_s_ recovery being more delayed than *A*_sat_ in most species. Furthermore, none of the six species exhibited significant hydraulic recovery during the 7 days re-watering period, indicating that xylem refilling was apparently limited; in addition, NSC availability had a minimal role in facilitating hydraulic recovery during this short-term period. Collectively, if water supply is limited by insignificant hydraulic recovery post-drought, the observed carbon assimilation recovery of seedlings may not be sustained over the longer term, potentially altering seedling regeneration and shifting forest species composition in subtropical China under climate change.

## Introduction

Forests contain almost 50% of terrestrial ecosystem carbon and thus represent a large carbon sink (Anderegg et al., 2015). Climate extremes, such as severe droughts, are predicted to occur more frequently with climate change (IPCC, 2021) and they may lead to forest die-off events, potentially impacting global carbon, water and energy balances as well as ecosystem services (Breshears and Allen, 2002; Bonan, 2008; Anderegg et al., 2013a). Tree species across biomes worldwide may be equally vulnerable to hydraulic failure due to a small “hydraulic safety margin” (Choat et al., 2012, 2018). Thus, the ability to maintain xylem water transport during drought, and to recover xylem hydraulic function post-drought, is crucial for tree growth and survival (Zwieniecki and Secchi, 2015) in the context of climate change (Anderegg and Venturas, 2020). Given the importance of hydraulic recovery on tree carbon gain and forest resilience from multiple droughts (Anderegg et al., 2013b; Creek et al., 2018), improved understanding of the capacity to restore hydraulic function post-drought, and traits related to the rate of recovery across species or functional types, will enhance the prediction of forest responses to variable precipitation patterns.

Water is transported under tension (i.e., negative pressure) from roots to stomata in leaves mainly *via* xylem, according to the “cohesion-tension” theory (Dixon and Joly, 1895). When the soil or air dries, then air bubbles generated by increasing tension may spread among xylem conduits, block water transport and lead to reduced hydraulic conductivity, further causing xylem embolism and hydraulic failure (McDowell et al., 2008; Urli et al., 2013). Despite some degree of stomatal regulation at an early drought stage, water loss through cuticles may continue even after stomatal closure (*g*_min_), and contribute to the formation of xylem embolism during extended drought (Duursma et al., 2019; López et al., 2021). Furthermore, declines in hydraulic conductivity can also cause partial or complete stomatal closure, inhibiting carbon assimilation and affecting non-structural carbohydrate (NSC) availability (Adams et al., 2017). Accordingly, based on this interrelated water-carbon relationship, tree species with varying hydraulic strategies (e.g., isohydric-anisohydric behavior) may differ in carbon dynamics during drought stress (McDowell et al., 2008), which could also be present in species with contrasting leaf strategies (e.g., evergreen/deciduous) given the possible difference in hydraulic structure and carbon assimilation and storage traits (Choat et al., 2005; Galle et al., 2011; Yin and Bauerle, 2017). Nonetheless, there is still limited knowledge on how tree species with different leaf habits might differ in response to drought stress given the inter-connected relationship between water relations and gas exchange or NSC availability.

Hydraulic recovery can be facilitated *via* the formation of new xylem (Brodribb et al., 2010), which is the most likely pathway to fully restore hydraulic function post-drought, but it is a slow and long-term process (Choat et al., 2018). Alternatively, hydraulic recovery may occur more rapidly by refilling of embolized conduits. Xylem refilling may occur *via* root/stem positive pressure in saturated soil during low transpiration conditions (Hao et al., 2013; Choat et al., 2018), or through an active osmotic process under tension (i.e., novel refilling), whereby osmotic gradients generated by soluble sugars released from xylem parenchyma/phloem drive water droplets into embolized conduits (Salleo et al., 2009; Zwieniecki and Holbrook, 2009; Brodersen et al., 2010).

Several studies have observed that fast xylem recovery occurred in many woody species after soil re-wetting or under tension, while the rate of hydraulic function restoration differs across species (Klein et al., 2018; Ruehr et al., 2019), depending on species-specific abilities to recover and the preceding drought severity (Li et al., 2021). Current research suggests that stem NSC and available water storage both correlate with species-specific responses in xylem recovery. First, tree species with faster rates of xylem recovery have been found to exhibit higher stem NSC availability (particularly for soluble sugars) at the end of drought (Savi et al., 2016; Tomasella et al., 2019a,b) and larger changes in soluble sugars after drought (Tomasella et al., 2019b; Trifilo et al., 2019; Zeppel et al., 2019). Second, a recent framework hypothesized that species with high risk hydraulic strategies (e.g., high water use, low xylem embolism resistance, low or negative hydraulic safety margin) are likely to have greater capacities to repair embolized xylem (Klein et al., 2018). In support of this hypothesis, some studies report that tree species with lower embolism resistance displayed lower wood density and/or higher stem hydraulic capacitance and faster rate of xylem recovery (Ogasa et al., 2013; Trifilò et al., 2015).

Higher stem hydraulic capacitance has been suggested to facilitate xylem recovery (Johnson et al., 2012; Trifilò et al., 2015), mainly due to its possible correlation with larger amounts of live cells (e.g., parenchyma; Scholz et al., 2007). Higher capacitance could thus provide more water as well as NSC during recovery (Meinzer et al., 2009; Meinzer and McCulloh, 2013; Trifilò et al., 2015). However, knowledge gaps remain regarding the links between stem hydraulic recovery, NSC and hydraulic capacitance, among species (Klein et al., 2018; Tomasella et al., 2019c) or functional types (Kannenberg et al., 2019; Kannenberg and Phillips, 2019). In particular, potential differences in the patterns of water potential and gas exchange recovery after drought in tree species with different leaf habits, are not well understood (Mediavilla and Escudero, 2003; Galle et al., 2011; Yin and Bauerle, 2017).

During the soil re-wetting process, it is not clear how the rate of gas exchange recovery is coordinated with hydraulic recovery. Most studies have observed that gas exchange recovery lagged behind hydraulic recovery (Brodribb and McAdam, 2013; Martorell et al., 2014; Creek et al., 2018), but the degree was highly dependent upon the species (Duan et al., 2019; Ruehr et al., 2019). The divergent recovery of gas exchange among species post-drought has been found to be correlated to the hydraulic safety margin (Skelton et al., 2017). Species-specific differences in the rate of gas exchange recovery will have substantial impacts on forest growth and productivity post-drought. Therefore, enhanced understanding of gas exchange recovery across species or functional types will advance our ability to predict legacy effects of drought on the forest community.

The subtropical region in China exhibits a generally humid climate, but also experiences seasonal droughts or high temperature events. However, climate extremes have become more common over the last 5 years, including several severe droughts and heat waves (China Meteorological Administration, 2020). A recent study has revealed that the risk of flash drought (i.e., sudden severe drought) in the subtropical region of China is predicted to increase by 40% by mid-21st century (Yuan et al., 2019). Therefore, it is critical to investigate the drought resistance and resilience of tree species in this subtropical region. In this study, six broad-leaved tree species representing evergreen and deciduous leaf habits were chosen. The four evergreen species (*Cinnamomum camphora*, *Schima superba*, *Cyclobalanopsis glauca*, *Castanopsis sclerophylla*) are dominant tree species in forest ecosystems in subtropical China, while the two deciduous species (*Liquidambar formosana* and *Sapindus mukorossi*) co-occur in these forests. We examined morphological traits and a suite of physiological traits related to water and carbon relations during drought stress and subsequent recovery. Our objective was to compare responses across co-existing species, which differ in leaf habit, in the coordination of hydraulic function and gas exchange following re-watering after a drought event. We hypothesized that (i) species with faster rates of hydraulic recovery would display higher hydraulic capacitance and higher NSC availability at the end of drought stress; (ii) the four evergreen species would exhibit slower rates of hydraulic and gas exchange recovery than the two deciduous species, because evergreen species may have lower hydraulic capacitance (Choat et al., 2005; Yin and Bauerle, 2017); and (iii) *g*_s_ recovery would be related to hydraulic recovery.

## Materials and methods

### Plant material

One-year-old seedlings from each of the six species, *Cinnamomum camphora*, *Schima superba* Gardner & Champ., *Cyclobalanopsis glauca* (Thunb.) Oerst., *Castanopsis sclerophylla* (Lindl.) Schott., *Liquidambar formosana* Hance and *Sapindus mukorossi* Gaertn. were purchased from a local nursery. The first four tree species are dominant evergreen species in the evergreen broad-leaved forests in subtropical China, while the last two tree species are winter deciduous broad-leaved species which co-occur with these evergreen species (Shi et al., 2011; Gu et al., 2015; Zhu et al., 2018). In March 2018, sixty seedlings per species with similar heights and basal diameters (Table 1) were transplanted into 7.6 L pots with drainage holes at the bottom. Each pot contained one seedling and about 6 kg of air-dried red soil (Quaternary Red Earth). All pots were randomly placed in a rainout shelter (30 m length × 3 m width × 3 m height), with natural sunlight (*ca.* 15% of direct sunlight was reduced by the PVC roof; 28^o^41′17″N, 116^o^01′50″E). The annual mean precipitation in subtropical region of China generally ranges from 800 to 1,800 mm, with 1,700 mm in the place where this experiment was conducted. Besides, the wet season here is from April to October, while the dry season is from November to March. About 50% of the annual rainfall falls between April and June. During the experimental period, the average maximum and minimum temperatures were 21.3°C and 8.6°C, respectively, while mean relative humidity was 73%. Seedlings were initially well irrigated daily and fertilized every 2 weeks with a commercial fertilizer (Shikede Horticultural Fertilizer Co. Ltd., Wuhan, China; *N* ≥ 30 g/L, P_2_O_5_ ≥ 14 g/L, K_2_O ≥ 16 g/L, Fe ≥ 0.14 g/L, Mn ≥ 0.06 g/L).

**Table 1:** Plant traits for the tree species used in this study.

Species	Family	Leaf habit	*σ*	Height (cm)	Basal diameter (mm)	Dry mass (g)	Root to shoot ratio	Estimated *P*_50_ (MPa)	Achieved Ψ_xylem_ (MPa)	Achieved PLC (%)
*Cinnamomum camphora*	Lauraceae	Evergreen	0.96 (0.06)	50.0 (1.9)	7.9 (0.2)	23.7 (2.8)	2.0 (0.4)	−2.3 (−2.9, −1.9)	−2.8 (0.1)	59 (5)
*Schima superba*	Theaceae	Evergreen	1.14 (0.03)	38.6 (1.5)	8.7 (0.3)	25.9 (3.1)	1.1 (0.1)	−2.3 (−2.6, −2.2)	−3.7 (0.1)	65 (5)
*Cyclobalanopsis glauca*	Fagaceae	Evergreen	1.06 (0.05)	47.0 (1.4)	6.8 (0.2)	29.4 (4.2)	1.2 (0.3)	−2.4 (−2.9, −1.9)	−2.6 (0.2)	57 (10)
*Castanopsis sclerophylla*	Fagaceae	Evergreen	1.16 (0.07)	42.8 (1.9)	6.1 (0.5)	16.4 (2.6)	0.9 (0.1)	NA	−1.9 (0.1)	50 (2)
*Liquidambar formosana*	Hamamelidaceae	Deciduous	0.97 (0.08)	76.0 (3.6)	10.4 (0.3)	59.4 (6.8)	0.8 (0.1)	−2.3 (−2.7, −1.9)	−2.1 (0.2)	66 (4)
*Sapindus mukorossi*	Sapindaceae	Deciduous	1.03 (0.14)	107.5 (3.9)	13.6 (0.3)	67.7 (5.2)	1.2 (0.1)	−0.8 (−1.2, −0.6)	−2.4 (0.2)	70 (5)

Values are Means with SE or 95% CI in parentheses. *σ*, slope of Ψ_l_ vs Ψ_pd_, reflecting the degree of anisohydry: *σ* > 1, more anisohydric; *σ* < 1, more isohydric (see Martínez-Vilalta et al., 2014). Values of height and basal diameter (*n* = 21–30) and total dry mass (*n* = 6–9) represent values at the beginning of the experiment. Estimated P_50_ was derived from hydraulic vulnerability curves conducted at the beginning (*n* = 13–25). Achieved Ψ_xylem_ (*n* = 3–4) and PLC (*n* = 3–4) represent xylem water potential and PLC at peak drought stress, respectively. NA, data is not available.

### Water, drought and recovery treatments

Following 6 months of growth (September 2018), plant size and dry mass exhibited differences among species similar to the beginning of the experiment (Table 1) and did not vary much across the experimental period (data not shown). For a given species, seedlings were randomly assigned to each of the two watering treatments: well-watered treatment and drought-recovery treatment. Plants in the well-watered treatment (30 seedlings per species) were irrigated to field capacity throughout the experiment. By contrast, plants in the drought-recovery treatment (30 seedlings per species) received no water until the percentage loss of hydraulic conductivity (PLC) of the stem was *ca.* 50% (i.e., about 50% of hydraulic conductivity was lost) for a given species, representing a critical physiological condition as drought develops (Brodribb et al., 2020). Hence, seedlings from all species were subjected to a similar degree of biologically-relevant drought intensity; thus, results were comparable across species. We estimated the time to achieve the target PLC by monitoring the declines of xylem water potential (Ψ_xylem_) close to approximate *P*_50_ (i.e., the Ψ_xylem_ at which stem hydraulic conductivity was reduced by 50%) of a given species, which is estimated from hydraulic vulnerability curves using seedlings at the beginning of the experiment. The hydraulic vulnerability curves were conducted in five species except *C. sclerophylla* due to the small plant size of this species. Thus, we estimated its *P*_50_ referring to other evergreen species in this study. We acknowledge, in practice, that it is difficult to reach the exact target PLCs in the experiment, thus PLCs of some species exceeded 50%, but in the range of 50%–70% (Table 1). We checked if drought-stressed plants experienced a similar degree of water stress within species by randomly measuring the midday xylem water potential from five to nine plants. Thereafter, a subset of seedlings in the drought-recovery treatment were then re-watered to field capacity at 1800 h and soil water content was maintained at field capacity until the end of the experiment. We focused on the dynamic recovery of hydraulics and gas exchange; thus, post-drought measurements were taken at 1, 3 and 7 days after re-watering.

### Gas exchange

Throughout the experimental period, leaf gas exchange measurements were taken on current year, fully expanded leaves from four seedlings per treatment per species (*n* = 4) between 0900 h and 1100 h on clear days, using a portable open path gas exchange system (Licor-6,400, Li-Cor, Lincoln, NE, United States) equipped with a red-blue light source (6400-2B). Leaf photosynthesis under saturating light (*A*_sat_, μmol m^−2^ s^−1^) and stomatal conductance (*g*_s_, mol m^−2^ s^−1^) were measured at photosynthetic photon flux density (PPFD) of 1,500 μmol m^−2^ s^−1^, [CO_2_] of 400 μmol mol^−1^ and corresponding air temperature (25.6 ± 0.04°C). The leaf-to-air VPD was 1.8 ± 0.04 kPa across species. Leaf instantaneous water-use efficiency (*WUE*_i_, μmol mol^−1^) was calculated as *A*_sat_/*g*_s_.

### Water relations

Pre-dawn leaf water potential (Ψ_pd_, MPa), midday leaf water potential (Ψ_l_, MPa) and xylem water potential (Ψ_xylem_, MPa) were measured on four seedlings per treatment per species (*n* = 4) at each sampling date during the dry-down and recovery processes, using a Scholander-type pressure chamber (PMS 1505D, PMS instruments, Corvallis, Oregon, United States). On the evening prior to measurements, seedlings were randomly selected and each sampled seedling was double-bagged in a plastic bag overnight to ensure equilibration of the water potential between the soil and the seedling. Approximately 1 h before sunrise, Ψ_pd_ was measured. During the midday, leaves were sampled for the determination of Ψ_l_. For midday Ψ_xylem_ measurements, leaves were wrapped with plastic film and foil for at least 1 h before Ψ_xylem_ was estimated. We determined *σ* (the slope of Ψ_l_ vs Ψ_pd_), which reflects the relative degrees of anisohydry vs. isohydry. In particular, *σ* > 1 indicates a more anisohydric behavior, while *σ* < 1 reflects a tendency for isohydry (Martínez-Vilalta et al., 2014).

### Hydraulics

Shoots of three to four seedlings per water treatment per species were sampled for hydraulic measurements at predawn at each sampling date (i.e., peak drought and 1, 3, 7 days after re-watering). Stems were cut underwater close to the soil, wrapped with parafilm and sealed in a black plastic bag with wet paper towels to prevent evaporation. Samples were placed in the ice box and returned immediately to the laboratory. The initial Ψ_xylem_ was immediately measured using two leaves selected from the shoots. The cut ends of the sampled shoots were recut under water and entire shoots were covered in a black plastic bag until Ψ_xylem_ was >−0.5 MPa (Torres-Ruiz et al., 2015; Creek et al., 2018), ensuring relaxation of the xylem tension within the stem and avoiding possible excision artifacts (Wheeler et al., 2013; Torres-Ruiz et al., 2015). The shoot was then progressively cut back under water until a straight, unbranched stem segment ~15 cm in length was obtained.

Stems were recut immediately under KCl solution (2 mM) to prevent air entry into the xylem and stem segments of 7–10 cm in length were then cut for hydraulic measurements. The two ends of the segments were shaved smoothly with a razor. With a pressure head of about 5.4 kPa (i.e., 54 cm of height), the initial/pre-flush hydraulic conductivity (*K*_initial_) was estimated from the segment by measuring the flow rate of KCl solution using a XYL’EM device (Xylem Embolism Meter, Bronkhorst, Montigny les Cormeilles, France). This pressure head is similar to that in other studies (Hao et al., 2011; Li et al., 2015; Gong et al., 2021). *K*_initial_ was calculated as the ratio of flow rate through the stem segment and the pressure gradient generating the flow. Then, the same segment was flushed with the same solution for 30 min under a pressure of 100 kPa to remove any embolism to establish *K*_max_. The percentage loss of conductivity (PLC) of the stem segment was determined by:


(1) $$\mathrm{PLC}=\frac{K_{\max}-K_{\mathrm{initial}}}{K_{\max}}100$$

In addition, hydraulic vulnerability curves were conducted at the beginning of the experiment using 13–25 seedlings per species from the well-watered treatment, following the bench drying method (Sperry et al., 1988). Harvested seedlings were dehydrated on the bench and one seedling was chosen for hydraulic measurement each time during the dry-down process. Values of PLC and Ψ_xylem_ were measured overtime and the hydraulic vulnerability curves were generated by plotting PLC against Ψ_xylem_.

Huber value (*HV*, ×10^−4^) was calculated as sapwood area (*A*_s_) divided by the supported leaf area (*A*_l_). The *A*_s_ was estimated from the area of sample cross sections by taking two diameter measurements (from different directions) and averaging values, using digital calipers. The seedlings did not have much pith or bark, so we did not need to accommodate this issue, although that must be done for older trees (Li et al., 2015; López et al., 2021). The *A*_l_ was measured using a portable leaf area meter (Licor-3100A, Li-Cor Inc. United States).

### Hydraulic capacitance

Pre-drought, whole branch hydraulic capacitance (*C*_branch_) was measured on well-watered plants using the bench dehydration method described previously by Li et al. (2018). The plant above the root collar was cut predawn and covered by a black plastic bag. Branches were placed in the ice box and were then taken to the laboratory. In the laboratory, branches were first rehydrated underwater for 12 h before the fresh branch weight and Ψ_xylem_ were measured. Branches were then allowed to dehydrate on the bench, during which time repeated measurements of Ψ_xylem_ and branch fresh weight were conducted. At each step, the branch was placed into a sealed plastic bag for roughly 1 h to ensure equilibration between Ψ_l_ and Ψ_xylem_. When these measurements were completed, branch samples were oven-dried at 70°C for 72 h to determine the dry mass. Branch relative water content (RWC_branch_, %) at each step was plotted against Ψ_xylem_. *C*_branch_ was estimated as the slope of the second linear portion of the water release curve normalized by shoot dry mass, expressed as ΔRWC/(ΔΨ × Δdry mass) (RWC% MPa ^−1^ g^−1^; Li et al., 2018).

### Minimum leaf conductance (*g*_min_)

We estimated pre-drought minimum leaf conductance (*g*_min_; i.e., the rate of water loss through the cuticle after stomata are closed; see Duursma et al., 2019) from two detached leaves from each of three to four randomly selected well-watered seedlings per species, following the method of Sack and Scoffoni (2011). Leaves were scanned for leaf area (Licor-3100A, Li-Cor Inc. United States) and then dried in a growth chamber, with the air temperature of 25°C, relative humidity of 50%–60% and a light intensity of <5 μmol m^−2^ s^−1^. Leaves were then weighed every 20 min over the period of 120–300 min, depending on species, using a high precision balance. Leaf *g*_min_ (mmol m^−2^ s^−1^) was calculated from the slope of the linear part of leaf mass vs. time regression in conjunction with chamber VPD and leaf area.

### Growth and leaf carbon isotopic composition

Plant height (*H*, cm) and basal diameter (*D*, mm) were measured regularly over the experimental period. Basal diameter was measured at 5 cm above the soil. The plants used for hydraulic measurements were harvested and we separated the leaves, stems and roots, which were washed free of soil. All harvested fresh organs were placed into an oven at 110°C for 1 h to eliminate biological activity and then oven-dried at 70°C for 72 h for dry mass determination. Leaf area (cm^2^) was determined using a portable leaf area meter (LI-3100A, Li-Cor, Lincoln, NE, United States). Specific leaf area (SLA, cm^2^ g^−1^) was calculated as the ratio of leaf area to leaf dry mass. Leaf carbon isotopic composition (δ^13^C, ‰) was measured on dried samples, using a PE2400 elemental analyzer (PerkinElmer, United States) connected to an IsoPrime100 isotope ratio mass spectrometer (Elementar, Germany). Leaf δ^13^C was used to estimate the integrated, long-term leaf water-use efficiency. One stem segment per harvested seedling was used to determine stem wood density (g cm^−3^). The volume of the fresh segment (without bark) was determined gravimetrically by the water displacement method. The dry mass was measured after 72 h by oven-drying at 70°C. Wood density was calculated as the stem dry mass divided by the stem volume.

### Non-structural carbohydrate assay

After each harvest (i.e., pre-drought, peak drought and 1, 3, 7 days after re-watering), oven-dried plant organ samples were ground to fine powder in a ball mill and NSC concentrations were analyzed as detailed in Duan et al. (2019). Shortly, organ samples (50 mg) were weighed and then extracted with 4 ml of 80% aqueous ethanol (v/v) in a polyethylene tube. The mixture was boiled in a water bath at 80°C for 30 min, and then centrifuged at 3000 rpm for 5 min. The supernatant was collected and the pellet re-extracted once with 4 ml of 80% aqueous ethanol (v/v) and once with 4 ml of distilled water, then boiled and centrifuged as before. Total soluble sugars (Ss) and starch (St) were determined on the supernatants or pellets using the modified anthrone method (Ebell, 1969; Quentin et al., 2015). NSC was defined as the sum of starch and soluble sugars.

### Statistical analyses

All statistical analyses were conducted with the open-source statistical software platform R (version 4.0; R Foundation for Statistical Computing). Time-series of traits (i.e., water potential, *A*_sat_ and *g*_s_) were analyzed within each species using a two-way ANOVA, with water treatment and date included as fixed factors; plant number was not considered as a random factor because plants were randomly chosen each time. At each sampling date, a one-way ANOVA was used to compare the difference between water treatments. For the inter-species or drought stage comparison of traits (i.e., most analysis), a one-way ANOVA was conducted followed by a Tukey *post hoc* test. We ensured homoscedasticity and normality were met prior to all statistical analyses. Results were considered statistically significant at *p* ≤ 0.05.

In the hydraulic vulnerability curves, PLC against Ψ_xylem_ of a given species was fitted with a Weibull function using the *fitplc* package in R (Duursma and Choat, 2017) and *P*_50_ was then estimated. For each species, the response of *g*_s_ to increasing Ψ_xylem_ was fitted with a “sigmoidal” model in conjunction with the “fitcond” function in the *fitplc* package, wherein the Ψ_xylem_ at 80% stomatal closure (Ψ_*g*s80_) was estimated. Linear regressions among traits were also fitted in R.

## Results

### Water potential and gas exchange during drought and recovery

**Figure 1:**
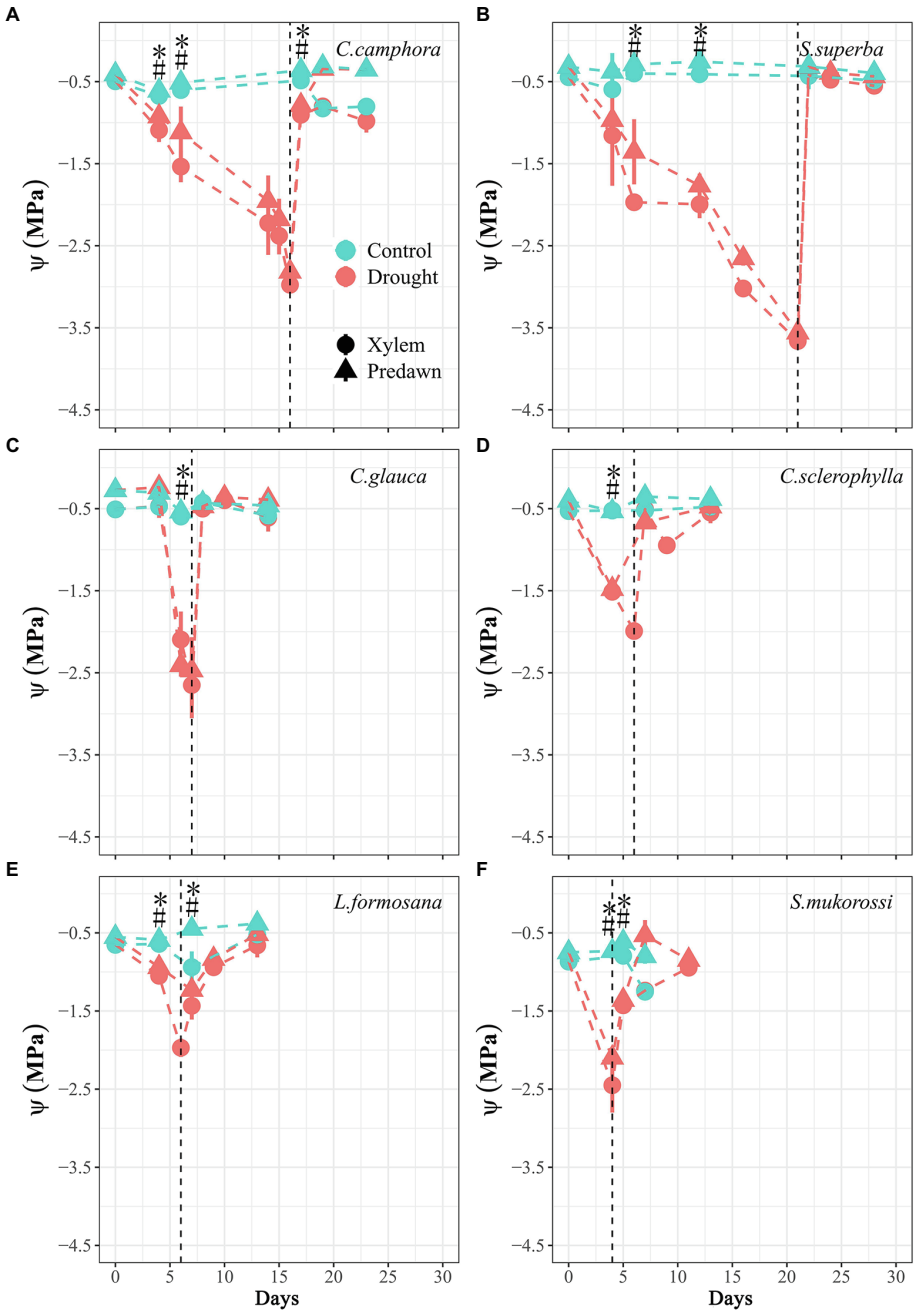
Time course of xylem water potential (Ψ_xylem_) and predawn water potential (Ψ_pd_) for seedlings of the six tree species throughout the experimental period. Values are Means ± SE (*n* = 3–5). “*” or “#” means significant difference (*p* ≤ 0.05) for Ψ_xylem_ or Ψ_pd_ between Control and Drought treatments at each sampling date. Control-Well-watered control; Drought-drought and recovery treatment. The vertical lines represent the time for re-watering. The first four species are evergreen species, while the last two species are deciduous species.

At the beginning, the two deciduous species *L. formosana* and *S. mukorossi* exhibited higher *g*_min_ than other species, but the four evergreen species did not have contrasting patterns of most economic and hydraulic traits compared with deciduous species (Table 2). During the dry-down process, the rate of decline in Ψ_xylem_ and Ψ_pd_ varied among species but not between evergreen and deciduous habits (Figure 1), with *S. mukorossi* exhibiting the most rapid decline and *C. camphora* displaying a slower decline. The different rates of declines in water potential among species was partly associated with the different plant sizes at the beginning (Table 1). The Ψ_xylem_ associated with target PLC level (*ca.* 50%), were observed after 4 days for *S. mukorossi* (Ψ_xylem_ = −2.4 MPa; PLC = 70%) and 6 days for *L. formosana* (Ψ_xylem_ = −2.1 MPa; PLC = 66%) in deciduous species; and 6 days for *C. sclerophylla* (Ψ_xylem_ = −1.9 MPa; PLC = 50%), 7 days for *C. glauca* (Ψ_xylem_ = −2.6 MPa; PLC = 57%), 16 days for *C. camphora* (Ψ_xylem_ = −2.8 MPa; PLC = 59%) and 21 days for *S. superba* (Ψ_xylem_ = −3.8 MPa; PLC = 65%) in evergreen species. After re-watering, the four evergreen species exhibited fast complete recovery of Ψ_xylem_ in 1 days, while the two deciduous species required 3 days to recover Ψ_xylem_ to control values (Figure 1).

**Table 2:** Plant economic and hydraulic traits for pre-drought seedlings of the six tree species.

Species	SLA (cm^2^ g^−1^)	Wood density (g cm^−3^)	*HV* (× 10^−4^)	*A*_sat_ (μmol m^−2^ s^−1^)	*g*_s_ (mol m^−2^ s^−1^)	*g*_min_ (mmol m^−2^ s^−1^)	δ^13^C (‰)	*C*_branch_ (RWC% Mpa^−1^ g^−1^)	Ψ_gs80_ (MPa)
*Cinnamomum camphora*	114.99b (4.43)	0.46d (0.01)	9.99bc (0.68)	12.68a (0.22)	0.21a (0.02)	1.73d (0.14)	−33.09cd (0.53)	0.34bc (0.02)	−1.0a (−1.3, −0.6)
*Schima superba*	80.20d (2.49)	0.48cd (0.01)	11.58bc (2.25)	7.01b (0.60)	0.11c (0.01)	3.28c (0.50)	−32.11c (0.21)	0.49b (0.11)	−2.2b (−2.6, −1.4)
*Cyclobalanopsis glauca*	80.39d (0.80)	0.64b (0.02)	5.9cd (1.25)	5.97b (1.39)	0.12c (0.03)	1.80d (0.54)	−31.47b (0.02)	0.54ab (0.06)	−1.5ab (−1.8, −1.1)
*Castanopsis sclerophylla*	79.40d (5.39)	NA	18.10ab (3.50)	6.76b (0.19)	0.17b (0.01)	3.03c (0.31)	−31.17b (0.26)	0.49b (0.10)	−1.3ab (−1.5, −0.9)
*Liquidambar formosana*	90.28c (3.78)	0.50c (0.01)	7.81cd (2.36)	3.10c (0.76)	0.10c (0.02)	8.99a (0.72)	−28.99a (0.07)	0.34bc (0.04)	−1.4ab (−1.7, −1.0)
*Sapindus mukorossi*	196.66a (16.28)	0.74a (0.03)	3.52d (0.73)	6.59b (0.66)	0.09c (0.01)	5.03b (0.35)	−32.11c (0.04)	0.05d (0.01)	−1.9b (−2.2, −1.4)

Values are Means with SE or 95% CI in parentheses. Different letters denote significant differences among species for a trait (*p* < 0.05). SLA, specific leaf area (*n* = 6–8); Wood density, stem wood density without bark (*n* = 8); HV, Huber value, the ratio of sapwood area to leaf area (*n* = 3–12); *A*_sat_, leaf photosynthesis under saturating light (*n* = 4); *g*_s_, leaf stomatal conductance (*n* = 4); *g*_min_, minimum leaf conductance (*n* = 6–8); leaf δ^13^C, leaf carbon isotopic composition (*n* = 3); *C*_branch_, normalized branch hydraulic capacity by shoot dry mass (*n* = 3–4); Ψ_gs80_, the water potential corresponding to 80% stomatal closure.

**Figure 2:**
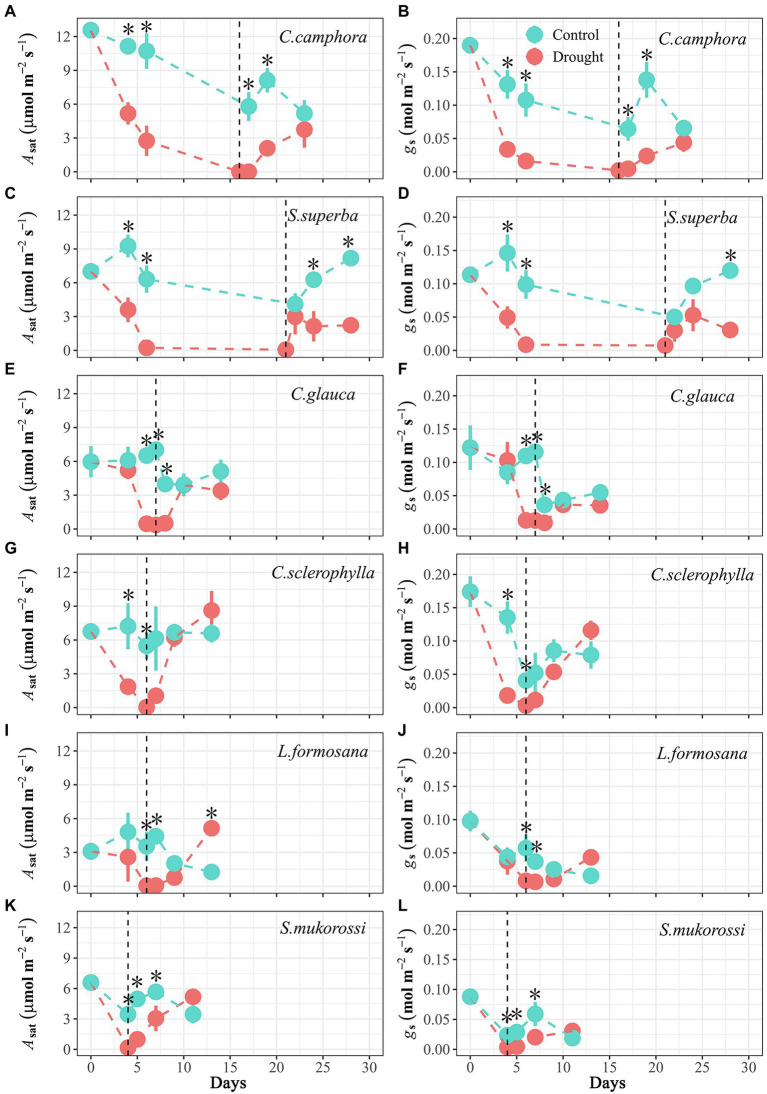
Time course of leaf photosynthesis under saturating light (*A*_sat_) and stomatal conductance (*g*_s_) for seedlings of the six tree species throughout the experimental period. Values are Means ± SE (*n* = 3–5). “*” means significant difference (*p* ≤ 0.05) for *A*_sat_ or *g*_s_ between Control and Drought treatments at each sampling date. Control-Well-watered control; Drought-drought and recovery treatment. The vertical lines represent the time for re-watering. The first four species are evergreen species, while the last two species are deciduous species.

Gas exchange (*A*_sat_ and *g*_s_) varied in accordance with water potential during the dry-down process (Figure 2). It took about 4–6 days for most species to achieve approximate zero in *A*_sat_ and *g*_s_, but longer for *C. camphora*. However, upon relief of drought stress, the rate of recovery in *A*_sat_ and *g*_s_ differed among species, but not between evergreen and deciduous groups (Figure 2). The evergreen *C. camphora* exhibited 30% recovery of *A*_sat_ and 19% recovery of *g*_s_ at 7 days after re-watering compared with pre-drought values (Figures 2A,B), whereas *S. superba* exhibited only 31% recovery of *A*_sat_ and 27% recovery of *g*_s_ at 7 days after re-watering (Figures 2C,D). The other two evergreen species (*C. glauca* and *C. sclerophylla*) recovered *A*_sat_ to pre-drought values at 3 days after re-watering, but *g*_s_ recovery largely lagged behind *A*_sat_ recovery (after 7 days), thereby leading to higher post-drought *WUE*_i_ than pre-drought *WUE*_i_ (Figure 2; Supplementary Figures S1, S2). By contrast, the two deciduous species (*L. formosana* and *S. mukorossi*) showed full recovery of *A*_sat_ at 7 days after re-watering, but only recovered *g*_s_ by 38%–44% (Figures 2I–L).

**Figure 3:**
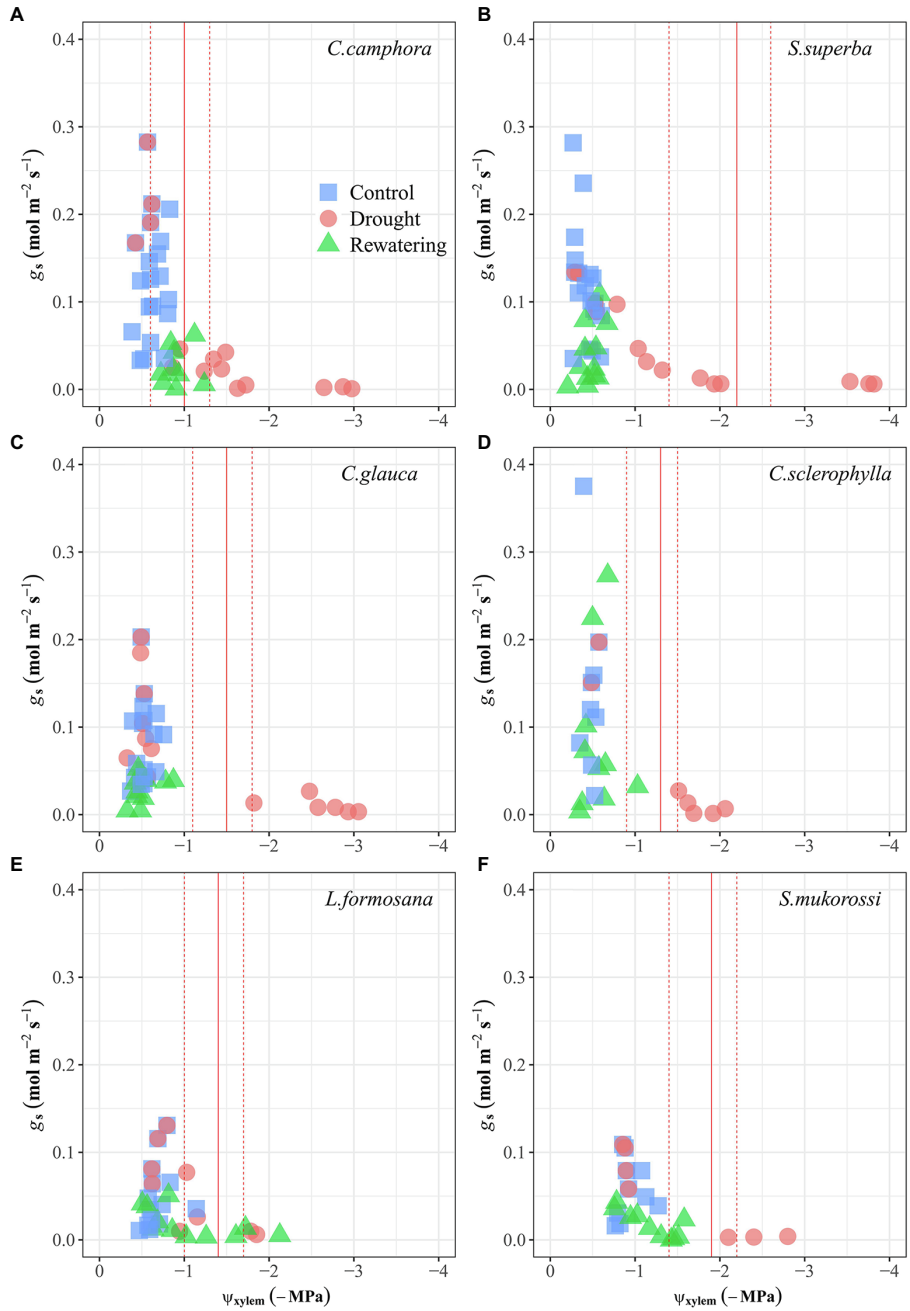
Leaf stomatal conductance (*g*_s_) as function of xylem water potential (Ψ_xylem_) for seedlings of the six tree species during drought and recovery periods. Values are raw points. Solid lines represent the Ψ_xylem_ for 80% loss of *g*_s_ (Ψ_*g*s80_), which were determined by the regressions fitted with a “sigmoidal” model. Dashed lines represent 95% confidence interval (CI). The first four species are evergreen species, while the last two species are deciduous species.

There was a marked hysteresis in the relationship of *g*_s_ and Ψ_xylem_ (Figure 3) between the dry-down and re-watering phases in most evergreen species (except *C. camphora*; Figures 3A–D), but the two deciduous species exhibited similar coordination of *g*_s_ and Ψ_xylem_ (Figures 3E,F). These species had similar *g*_s_ sensitivity to increasing drought stress (represented by similar Ψ_*g*s80_; Table 2; Supplementary Figure S3), which was also supported by convergence in the degree of isohydry (Table 1; Supplementary Figure S4).

### Hydraulics and NSC during drought and recovery

**Figure 4:**
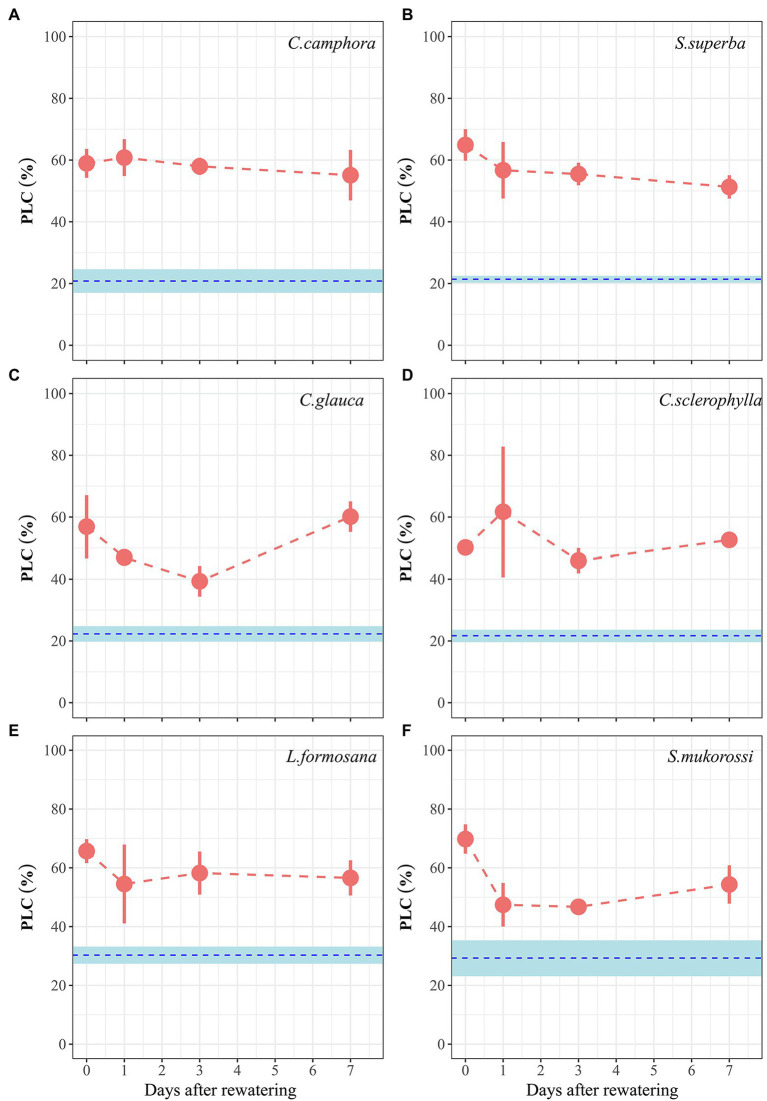
Time course of percentage loss of hydraulic conductivity (PLC) for seedlings of the six tree species during drought and recovery periods. Values are Means ± SE (*n* = 3–4). Red symbols represent PLC values in the Drought treatment. The blue dashed lines and shade areas refer to PLC values (with SE; *n* = 4–10) for each species grown in the well-watered control treatment throughout the experimental period. Day 0 represents peak drought stress and thus the time for re-watering. The first four species are evergreen species, while the last two species are deciduous species.

Well-watered plants of all species exhibited 20%–30% native PLC measured at predawn (Figure 4), with no difference between deciduous and evergreen species. At peak drought, mean PLC of the four evergreen species ranged from 50% to 65%, while those of the two deciduous species reached 66% (*L. formosana*) and 70% (*S. mukorossi*). The six species did not exhibit significant recovery of xylem hydraulic function during 7 days period of re-watering.

**Figure 5:**
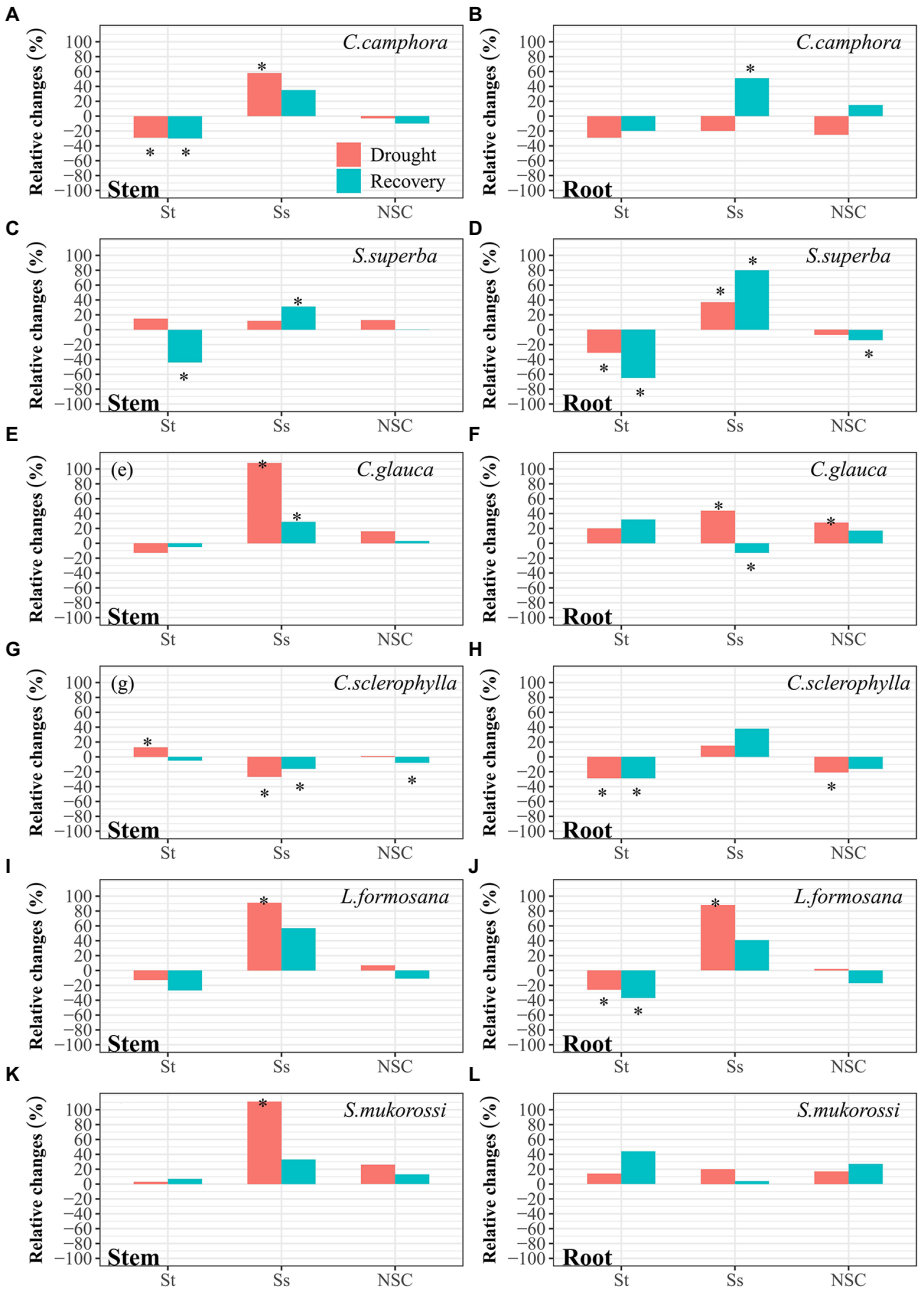
Relative changes of concentration of St, Ss and NSC in stems and roots for each species at peak drought (Drought) and 7 days after re-watering (Recovery) compared with pre-drought values. “*” means significant difference (*p* ≤ 0.05). St, starch; Ss, soluble sugars; NSC, non-structural carbohydrates. The first four species are evergreen species, while the last two species are deciduous species.

In the well-watered treatment, concentrations of starch (St), soluble sugars (Ss) and total NSC in stem and root for all species did not vary significantly throughout the experimental period (Supplementary Figures S5, S6). Collectively, organ NSC did not show significantly contrasting patterns between evergreen species and deciduous species. At pre-drought, *C. camphora*, *L. formosana* and *S. mukorossi* had higher stem NSC, while *C. camphora*, *S. superba* and *L. formosana* had higher root NSC. At peak drought, NSC varied significantly among organs and species (Figure 5). Compared to pre-drought values, stem St was reduced in *C. camphora*, but was elevated in *C. sclerophylla*. However, stem Ss increased in four species except *S. superba* and *C. sclerophylla*. Generally, stem total NSC did not change significantly in the species. Additionally, root St was decreased in *S. superba*, *C. sclerophylla* and *L. formosana*, while root Ss was increased in *S. superba*, *C. glauca* and *L. formosana*, leading to increased root NSC in *C. glauca* but reduced root NSC in *C. sclerophylla*. At the end of 7 days recovery period, NSC patterns varied depending on species. Reduction of stem NSC was evident in *C. glauca*, while reduction of root NSC was found in *S. superba*, mainly due to reductions in St.

## Discussion

### Water potential and gas exchange during drought and recovery

Whole plant performance during drought stress may be associated with a suite of hydraulic and morphological traits. During the dry-down process, reductions in plant water potential were significantly different between species, but not between the evergreen-deciduous groups. *S. mukorossi* had the most rapid decline in Ψ_xylem_ and Ψ_pd_ and highest peak drought PLC (70%), indicating faster drought responses compared to other species. Faster drought responses of *S. mukorossi* was a function of a suite of traits, including larger plant size, lower *HV*, lower *C*_branch_ and higher *g*_min_ (Supplementary Figure S7). First, larger plant size plus lower *HV* value reflect an imbalance between water demand and water supply (Ramírez-Valiente et al., 2020), thus facilitating an earlier response to drought stress. However, we have to be cautious with the plant size effect on whole plant water use when discussing drought responses among species, because species differ significantly in growth rates. Larger plants due to higher growth rates can consume greater amounts of water during drought stress, further increasing the risk of hydraulic dysfunction. Hence, some differences in drought responses among species may be related to plant size, in addition to differences in drought sensitivity of a given species. Second, despite similar drought sensitivities of *g*_s_ and *σ* values and convergent stomatal regulation strategies among the six species, *S. mukorossi* had higher *g*_min_ after stomatal closure, indicating greater water loss during extreme drought. Accordingly, *g*_min_ patterns could partly contribute to rapid water loss in this species, which further demonstrated the important role of *g*_min_ in regulating water loss in tree response to drought (Duursma et al., 2019).

When drought stress was relieved by re-watering, the six species exhibited fast recovery of Ψ_xylem_ in 1–3 days, which is mainly due to water movement in remaining functional conduits (Ruehr et al., 2019). However, we observed an *ca.* 2 days time lag between the four evergreen species and the two deciduous species, partially because the two deciduous species experienced greater drought stress associated with higher PLC (average of 68%) than the evergreen species (average of 58%). Accordingly, we suggest that less functional conduits due to higher drought-related PLC would hinder water transport required to recharge the leaf. Previous studies have also demonstrated that the time to recover physiology depends partially on the magnitude of the drought stress experienced (Resco de Dios et al., 2009; Brodribb et al., 2010; Tomasella et al., 2019b; Li et al., 2021).

The rate of recovery in leaf gas exchange differed significantly among species, but did not exhibit clear patterns between evergreen and deciduous groups. Evergreen *S. superba* and *C. camphora* did not fully recover *A*_sat_ and *g*_s_ during the 7 days re-watering period. However, other species exhibited full recovery of *A*_sat_, but complete or partial recovery of *g*_s_. These results demonstrate that there are different patterns of gas exchange recovery among species. Despite the important role of *g*_s_ on *A*_sat_ recovery from drought, most species exhibited more rapid recovery in *A*_sat_ than *g*_s_ and displayed high capacity to maximize leaf carbon assimilation at given water loss (Duan et al., 2019). The finding agrees with previous evidence that photosynthesis may exhibit faster recovery than stomatal conductance (Martorell et al., 2014; Creek et al., 2018), which could be related to rapid recovery of mesophyll conductance (Cano et al., 2014; Ruehr et al., 2019) or photosystem repair (Posch and Bennett, 2009; Sánchez-Gómez et al., 2013; Duan et al., 2019).

Additionally, we found that *g*_s_ recovery was decoupled from water potential recovery in half of the species. The decoupling of *g*_s_ and water potential during recovery from drought has been demonstrated in many studies, which may be mainly attributed to the accumulation of abscisic acid (ABA) in leaves during drought (Brodribb et al., 2014; Torres-Ruiz et al., 2014; Duan et al., 2020) and/or declines in leaf hydraulic conductance (Blackman et al., 2017; Creek et al., 2018). Thus, species may have contrasting patterns of ABA dynamics or leaf hydraulic conductance recovery after drought stress, that affects the rate of stomatal opening following re-watering. Future quantitative analysis of ABA dynamics and leaf hydraulic conductance will provide more insights into the mechanism underlying delayed gas exchange recovery from drought.

### Hydraulics during drought and recovery

During the dry-down process, PLC in all species was 50%–70%, which was approximately the target PLC of 50%. Upon re-watering, none of the six species significantly restored hydraulic function during the 7 days recovery period, suggesting that the capacity to restore hydraulic function was limited in these species (see reviews Klein et al., 2018; Ruehr et al., 2019) and fast hydraulic recovery may not be common (Choat et al., 2018). Moreover, we did not observe clear differences in the capacity to restore hydraulic function between functional types (i.e., evergreen and deciduous), which contrasts with our hypothesis. Based on earlier studies, deciduous species were hypothesized to have more axial parenchyma cells and water-carbon resources in the sapwood than evergreen species (Choat et al., 2005; Yin and Bauerle, 2017), thereby potentially enhancing physiological recovery post-drought. However, none of the deciduous species in our study were shown to display significant hydraulic recovery, thus rejecting our hypothesis. However, future studies are required to compare more tree species representing deciduous and evergreen growth forms to test the generality of our observation. We suggest that short-term xylem refilling may be a limited way to restore xylem function in many species and long-term re-growth of xylem may be required to fully restore hydraulic function (Brodribb et al., 2010; Choat et al., 2018; Gauthey et al., 2022).

The mechanisms underlying differences in hydraulic recovery among species remains inconclusive (Klein et al., 2018). Given the possible role of NSC in hydraulic recovery *via* osmotic processes, some studies have found that the species-specific rate of hydraulic recovery is positively correlated with stem NSC availability (primarily soluble sugars) at peak drought (Savi et al., 2016; Trifilò et al., 2017; Tomasella et al., 2019a). Other studies have shown that hydraulic recovery is associated with NSC utilization during recovery periods (Tomasella et al., 2017; Zeppel et al., 2019). In this short-term study, with observed limited hydraulic recovery in all species, we did not find a significant correlation between NSC availability and its recovery of xylem function (Supplementary Figure S8). Recent evidence has demonstrated that hydraulic recovery is positively related to stem hydraulic capacitance (Trifilò et al., 2015), but we did not observe a significant role of *C*_branch_ on hydraulic recovery in our species (Supplementary Figure S8), indicating that *C*_branch_ may have more important roles in larger trees than seedlings. Collectively, this study suggests that the evidence for fast hydraulic recovery through xylem refilling is limited for our species. Overall, the role of xylem re-growth and xylem refilling from positive pressure to generate hydraulic recovery may be species-specific, requiring additional studies to assess the relative roles of these two mechanisms in a variety of tree species.

## Conclusion

This study investigated short-term drought impacts and subsequent recovery responses of seedlings from four evergreen and two deciduous species by linking physiological and morphological traits, including the role of NSC and hydraulic structure, on the species-specific capacity to restore hydraulic function following re-watering. We did not find clear differences in hydraulic and gas exchange responses to increasing drought stress, as well as post-drought recovery, between deciduous and evergreen species; most differences were species-specific and potentially related to plant size. Upon relief of drought, none of the six species exhibited significant hydraulic recovery, which do not support the osmotic pressure xylem refilling mechanism driven by NSC and water source gradients. Despite observed partial or full recovery in carbon assimilation among species, failure to achieve full hydraulic recovery from a common severe drought level occurred in most species. If water supply is limited by insignificant hydraulic recovery post-drought, the observed carbon assimilation recovery of seedlings may not sustain in the longer term It is noted that this study was conducted on seedlings grown in pots, which requires caution when extrapolating our results to larger trees grown in the field. We also note that a limitation in this study is the short experimental period and acknowledge that different responses may have occurred under longer duration droughts. However, we are available to determine the differences of drought responses and the capacity of recovery among species with this approach determine the differences of drought responses and the capacity of recovery among species with this approach, which was similarly used in other studies (e.g., Ogasa et al., 2013; Tomasella et al., 2019a). Furthermore, our highly mechanistic study linked carbon and water relations of seedlings during drought and following recovery from drought in tree species representing two leaf habits (evergreen and deciduous), which will be useful for seedling cultivation and forest management under future climates.

## Data availability statement

The original contributions presented in the study are included in the article/Supplementary material, further inquiries can be directed to the corresponding author.

## Author contributions

HD conceived the study. HD, DW, and NZ conducted the experiment. HD, DW, GH, VR, and DT analyzed the results. HD wrote the manuscript with substantial input from GH, VR, and DT. All authors contributed to the article and approved the submitted version.

## Funding

This work was supported by grants from the National Natural Science Foundation of China (31600483 and 31760111) and the Natural Science Talent Funding of Guizhou University (202132).

## Conflict of interest

The authors declare that the research was conducted in the absence of any commercial or financial relationships that could be construed as a potential conflict of interest.

## Publisher’s note

All claims expressed in this article are solely those of the authors and do not necessarily represent those of their affiliated organizations, or those of the publisher, the editors and the reviewers. Any product that may be evaluated in this article, or claim that may be made by its manufacturer, is not guaranteed or endorsed by the publisher.
