# Supplementary material for: Limited hydraulic recovery in seedlings of six tree species with contrasting leaf habits in subtropical China
Source: Front Plant Sci. 2022 Aug 11;13:967187. doi: 10.3389/fpls.2022.967187 (PMC9403191; doi:10.3389/fpls.2022.967187)

Figure S1 Time course of leaf instantaneous water-use efficiency (*WUE*i) for seedlings of the six tree species throughout the experimental period. Values are Means ± SE (n=3-5). Control-Well-watered control; Drought-drought and recovery treatment. The vertical lines represent the time for re-watering. The first four species are evergreen species, while the last two species are deciduous species.

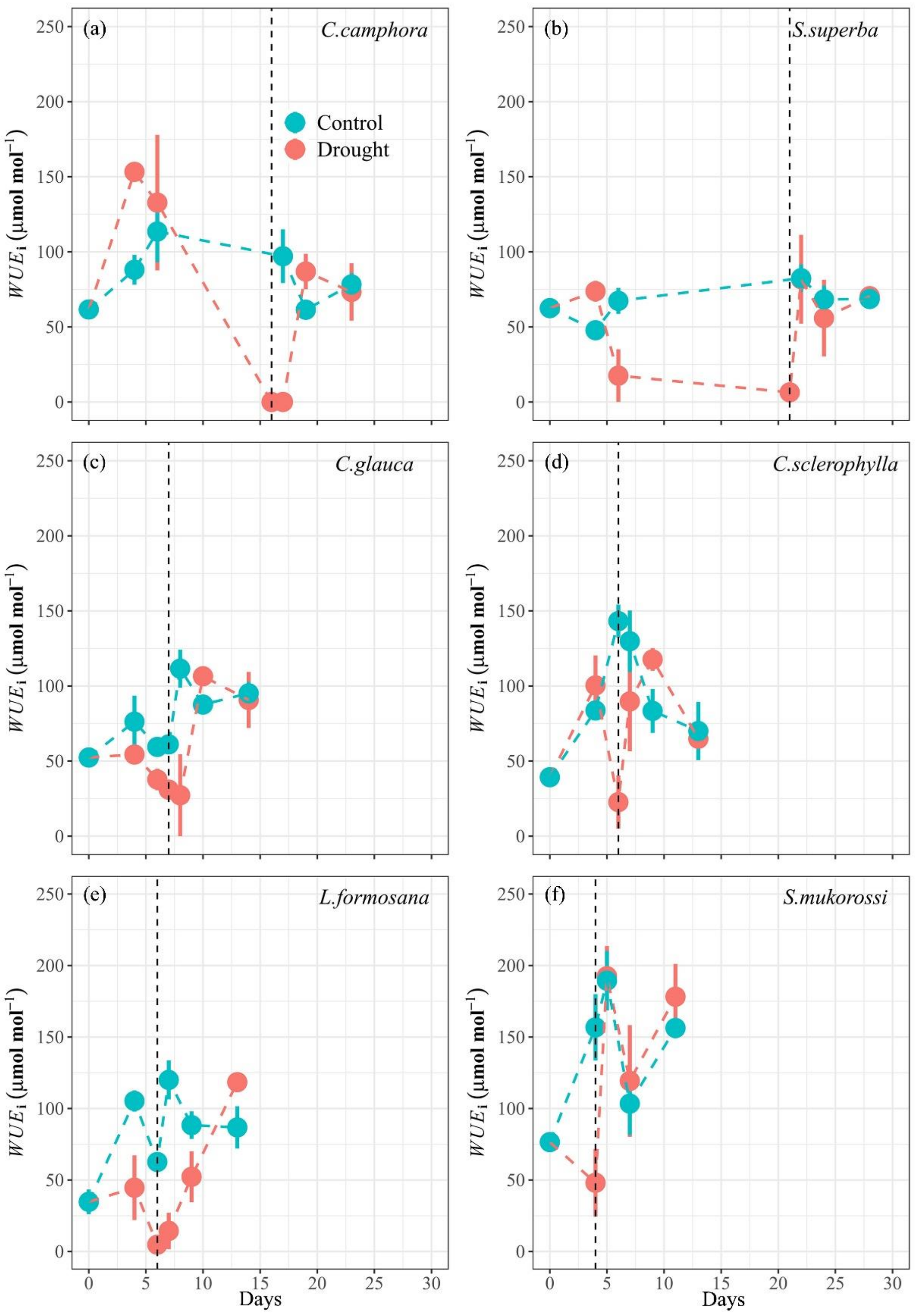

Figure S2 Leaf photosynthesis under saturating light (A_{sat}) as function of stomatal conductance (g_s) of seedlings of the six tree species during drought and recovery periods. Values are raw points.

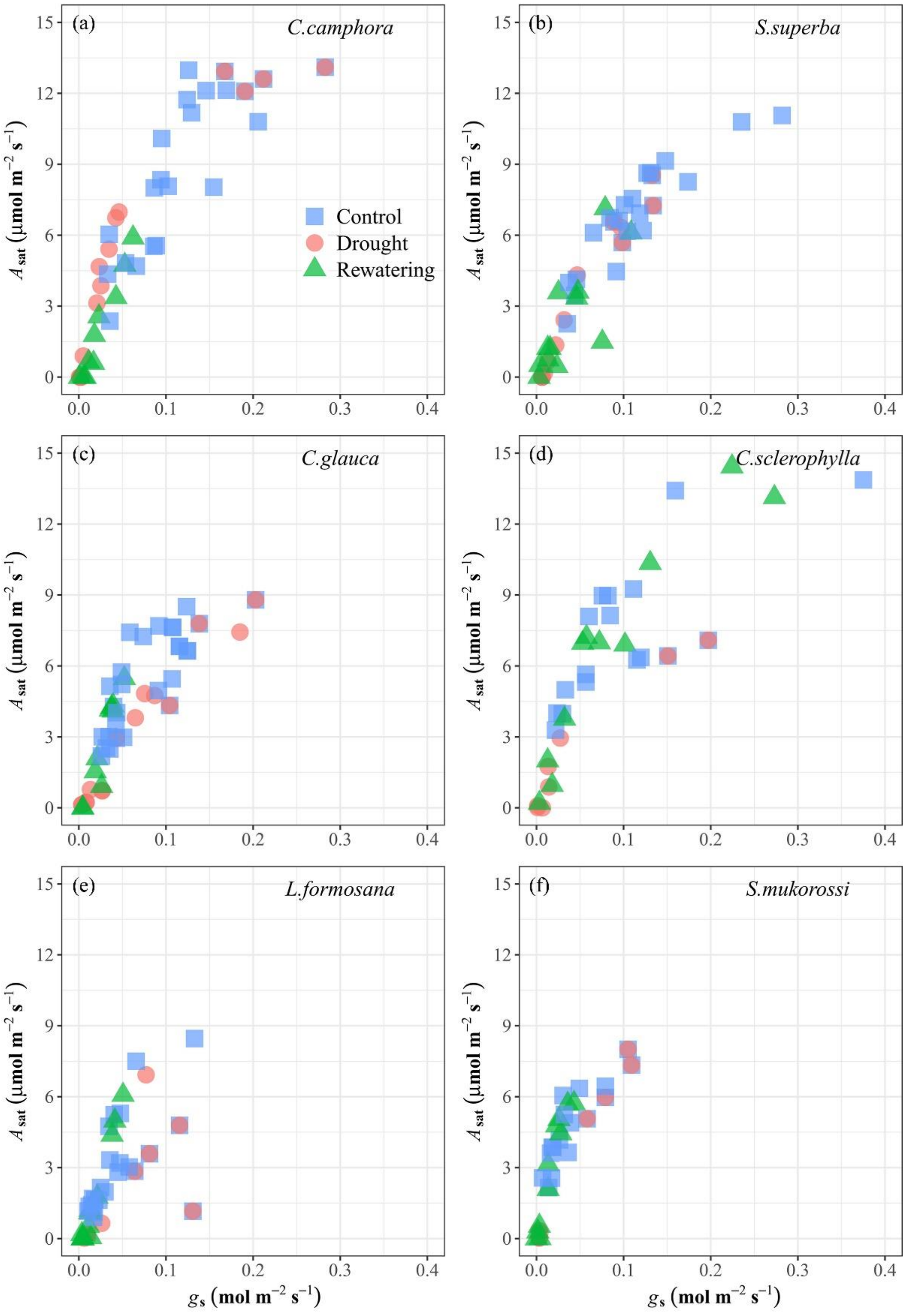

Figure S3 Leaf stomatal conductance (g_s) as function of xylem water potential (Ψ_{xylem}) for seedlings of the six tree species during drought period. Values are raw points. Solid lines represent the Ψ_{xylem} for 80% loss of g_s (Ψ_{gs80}), which were determined by the regressions fitted with a "sigmoidal" model. Dashed lines represent 95% confidence interval (CI) of Ψ_{gs80}. The shaded areas represent CI of fitted curves.

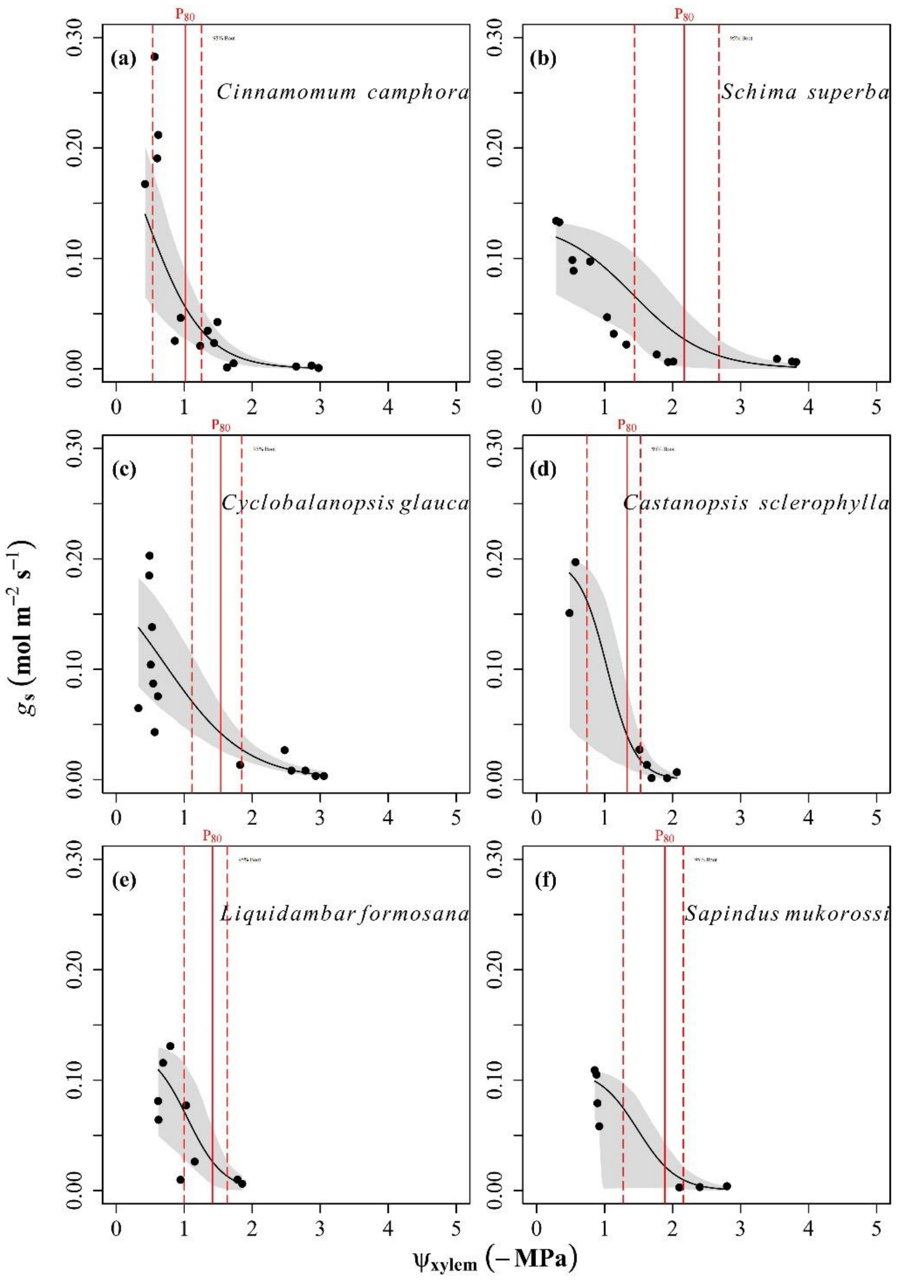

Figure S4 Midday leaf water potential (Ψ_l) as function of predawn leaf water potential (Ψ_{pd}) of seedlings of the six tree species during drought and recovery periods. Values are raw points.

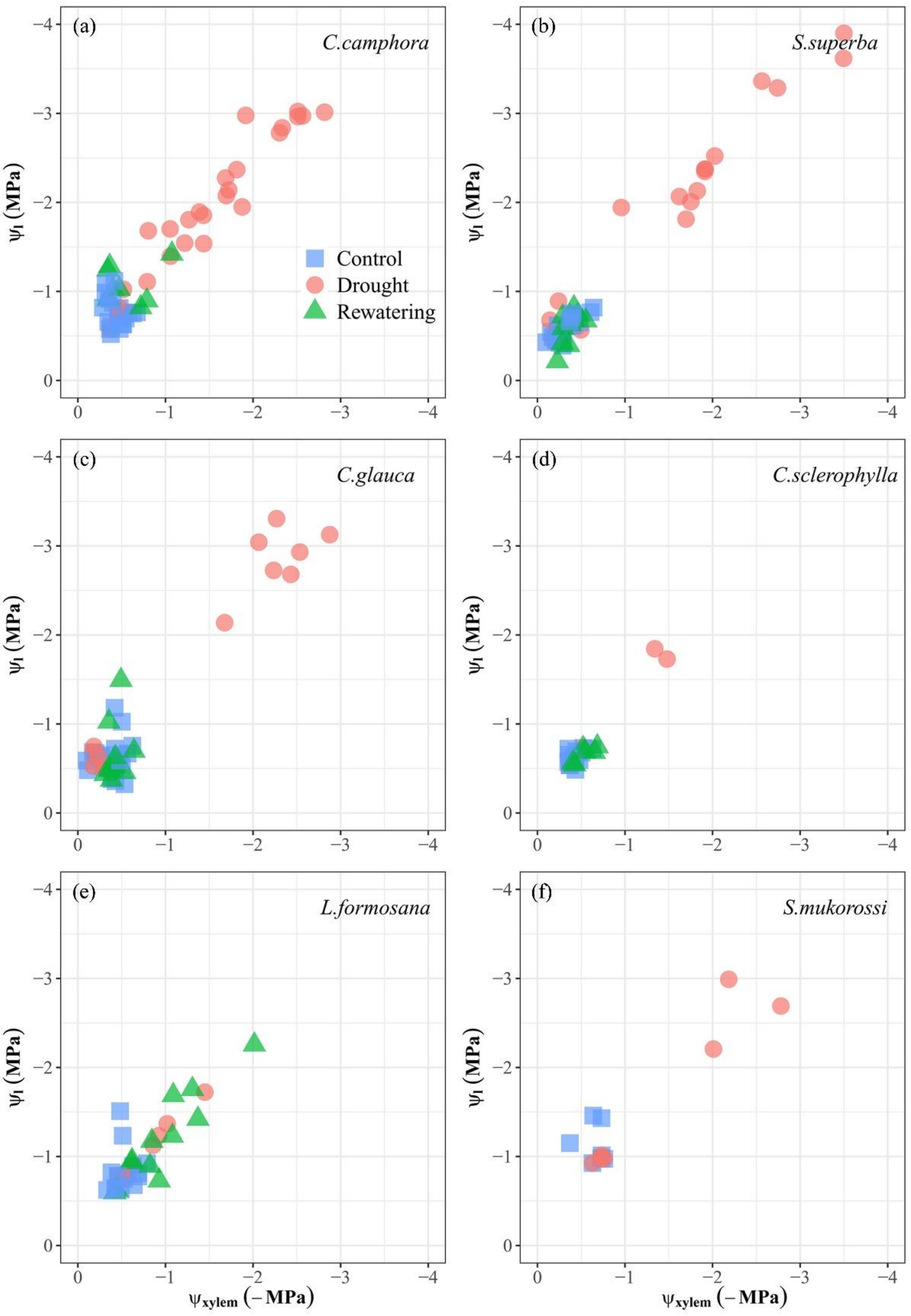

Figure S5 Time course of concentration of stem starch (St), soluble sugars (Ss) and NSC for seedlings of the six tree species throughout the experimental period. Values are Means ± SE (n=3-4).

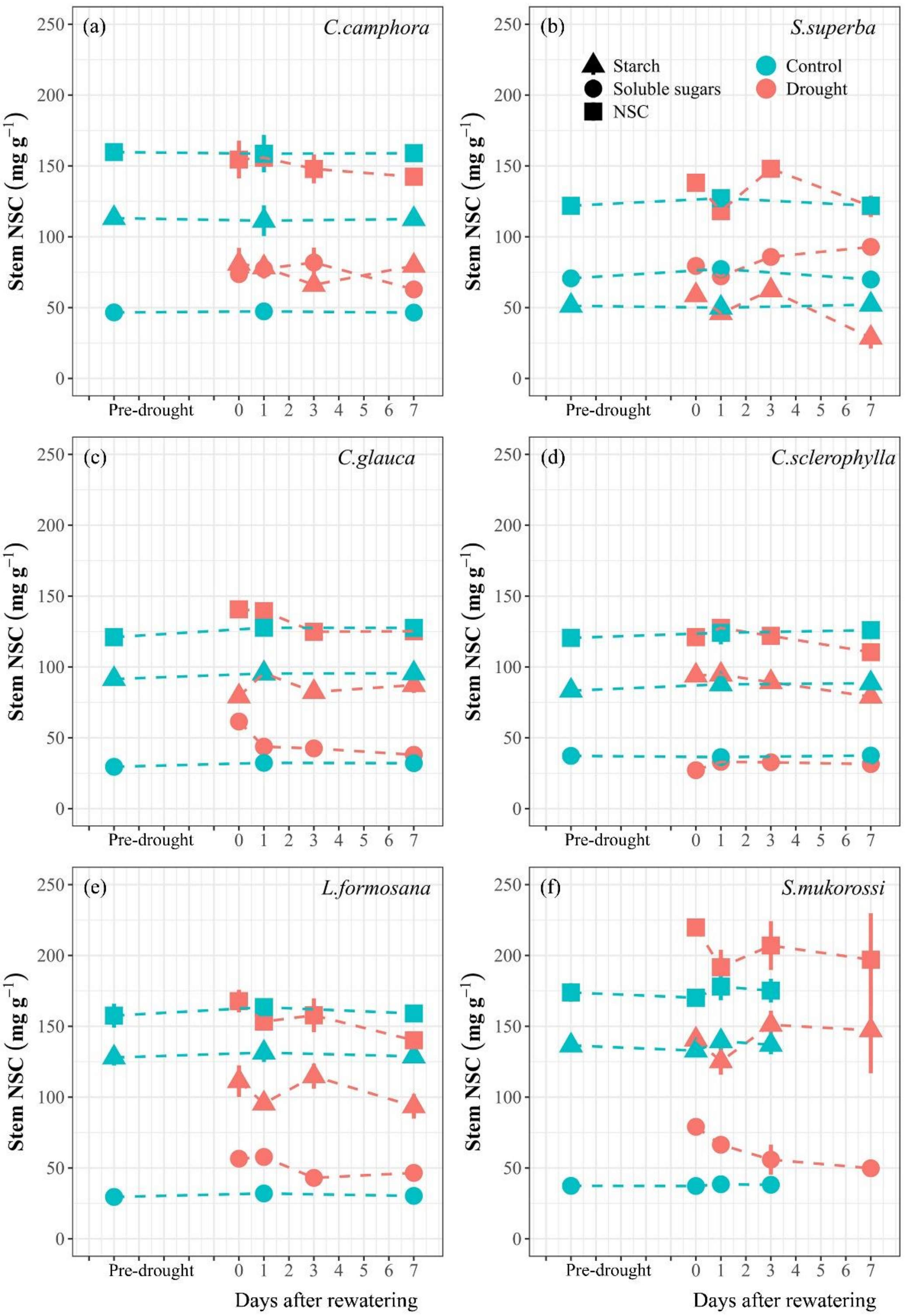

Figure S6 Time course of concentration of root starch (St), soluble sugars (Ss) and NSC for seedlings of the six tree species throughout the experimental period. Values are Means ± SE (n=3-4).

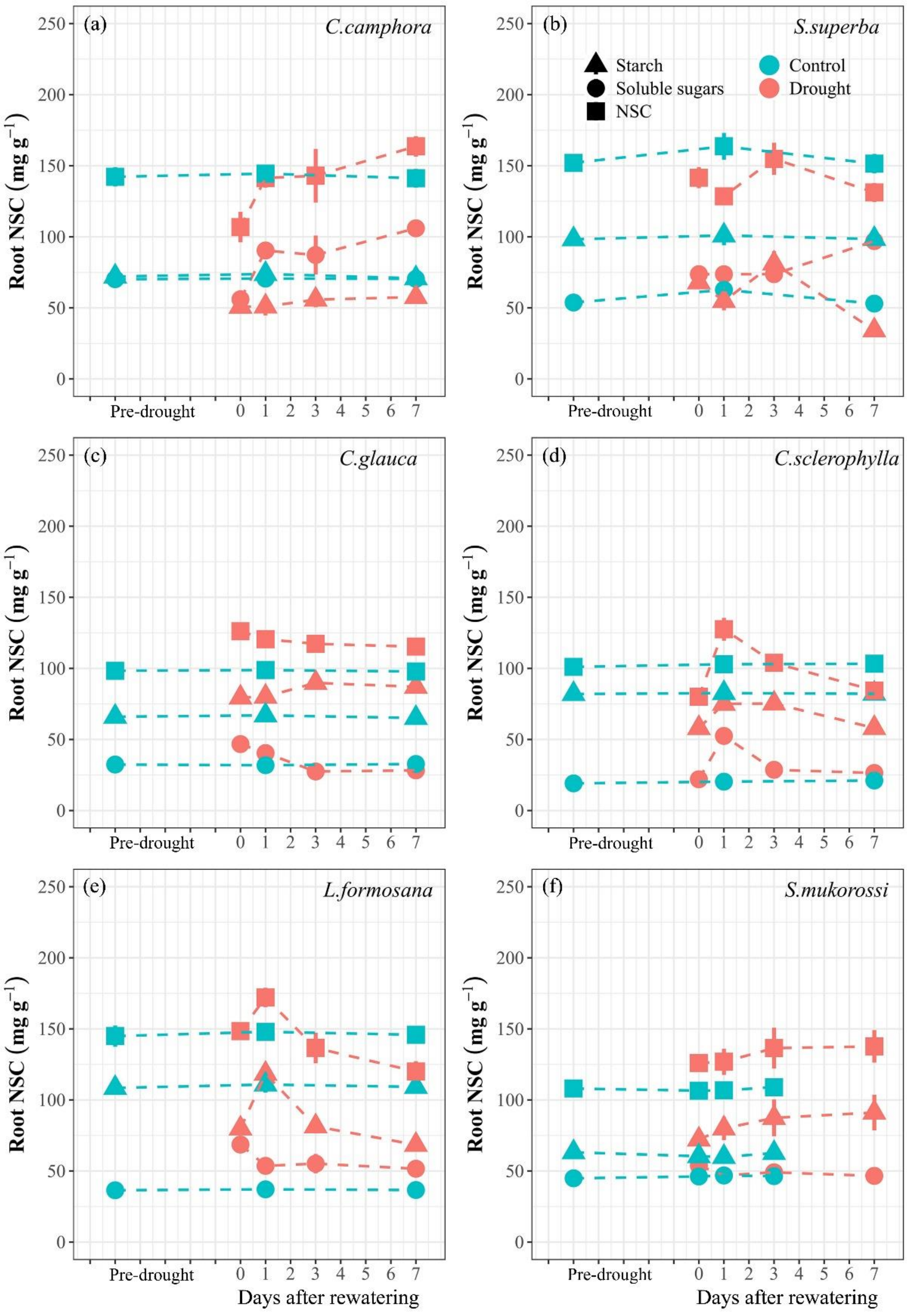

Figure S7 Principal components analysis (PCA) of four traits at predrought among the six species studied (a), showing the first principal component (PC1) explained 70.2% of total variance, while the second principal component (PC2) explained 19.2% of total variance. The factor loadings are shown as color gradients. The correlations among these traits are also shown in (b) and (c). Drymass, total dry mass; *HV*, Huber value, the ratio of sapwood area to leaf area; g_{min}, minimum leaf conductance; C_{branch}, normalized branch hydraulic capacity by shoot dry mass.

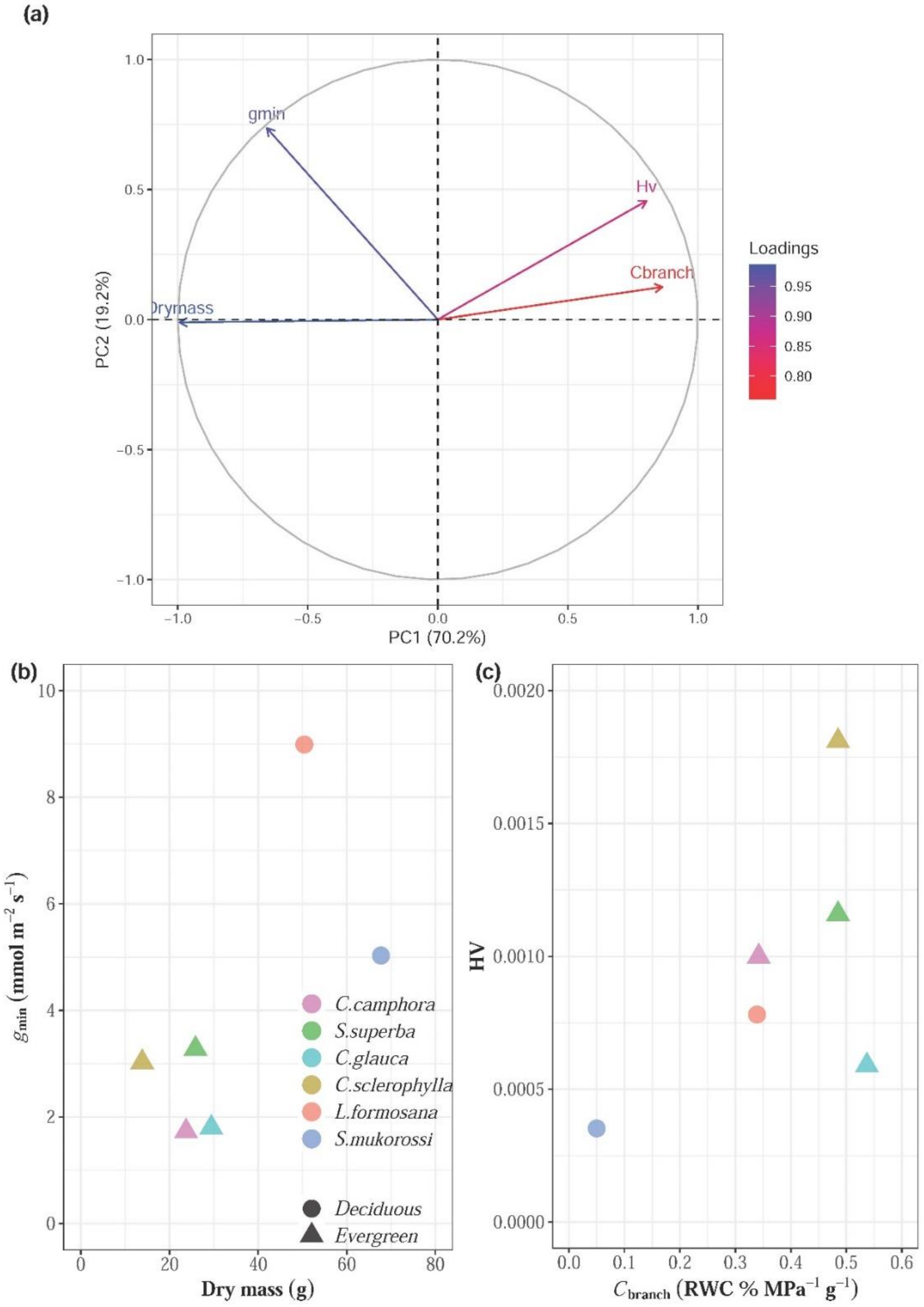

Figure S8 Relationships among traits for the six species. Leaf photosynthesis under saturating light (A_{sat}) as function of stomatal conductance (g_s) (a), leaf photosynthesis under saturating light (A_{sat}) as function of SLA (b), PLC difference between well-watered and drought treatments (∆ PLC) as function of branch hydraulic capacitance (C_{branch}) (c), stem Ss changes (relative changes of soluble sugars between peak drought and pre-drought) (d), stem St changes (relative changes of starch between peak drought and pre-drought) (e) and stem NSC changes (relative changes of NSC between peak drought and pre-drought) (f).

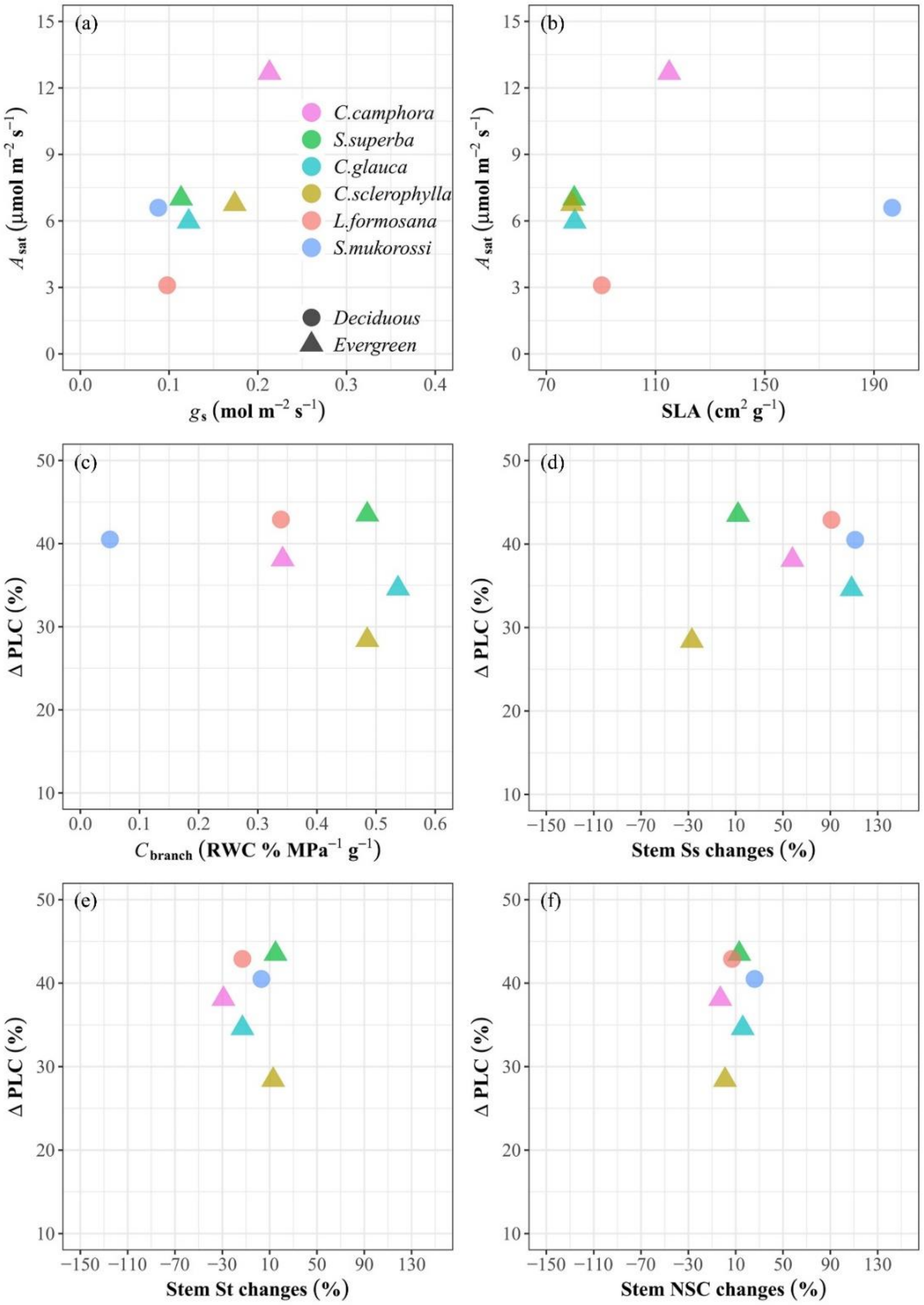

Figure S9 Stem hydraulic vulnerability curves for seedlings of studied species.

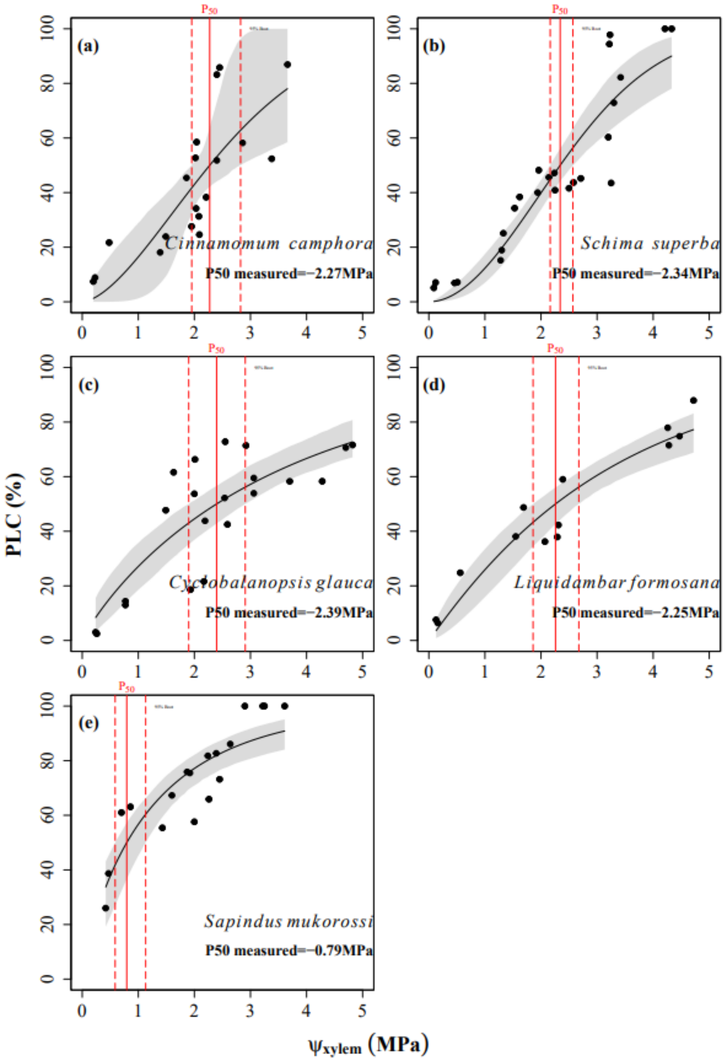

Supplement: Supplementary file 1 [file Presentation_1.pdf]
